# Micro Salting-Out Assisted Matrix Solid-Phase Dispersion: A Simple and Fast Sample Preparation Method for the Analysis of Bisphenol Contaminants in Bee Pollen

**DOI:** 10.3390/molecules26082350

**Published:** 2021-04-18

**Authors:** Jianing Zhang, Fengjie Yu, Yunmin Tao, Chunping Du, Wenchao Yang, Wenbin Chen, Xijuan Tu

**Affiliations:** 1 College of Food Science, Fujian Agriculture and Forestry University, Fuzhou 350002, China; zjnlo1225@163.com (J.Z.); yfj918@126.com (F.Y.); july766@126.com (Y.T.); beesyang@gmail.com (W.Y.); 2 College of Bee Science, Fujian Agriculture and Forestry University, Fuzhou 350002, China; du_cp39@163.com; 3 College of Animal Sciences, Fujian Agriculture and Forestry University, Fuzhou 350002, China

**Keywords:** sample preparation, matrix solid-phase dispersion, salting-out, homogenous liquid-liquid extraction, bisphenol, bee pollen

## Abstract

In the present work, a novel sample preparation method, micro salting-out assisted matrix solid-phase dispersion (μ-SOA-MSPD), was developed for the determination of bisphenol A (BPA) and bisphenol B (BPB) contaminants in bee pollen. The proposed method was designed to combine two classical sample preparation methodologies, matrix solid-phase dispersion (MSPD) and homogenous liquid-liquid extraction (HLLE), to simplify and speed-up the preparation process. Parameters of μ-SOA-MSPD were systematically investigated, and results indicated the significant effect of salt and ACN-H_2_O extractant on the signal response of analytes. In addition, excellent clean-up ability in removing matrix components was observed when primary secondary amine (PSA) sorbent was introduced into the blending operation. The developed method was fully validated, and the limits of detection for BPA and BPB were 20 μg/kg and 30 μg/kg, respectively. Average recoveries and precisions were ranged from 83.03% to 94.64% and 1.76% to 5.45%, respectively. This is the first report on the analysis of bisphenol contaminants in bee pollen sample, and also on the combination of MSPD and HLLE. The present method might provide a new strategy for simple and fast sample preparation of solid and semi-solid samples.

## 1. Introduction

In the past decade, matrix solid-phase dispersion (MSPD) has achieved great progress in the sample preparation of complex samples [1,2,3,4]. The major merit of MSPD is the accomplishing of extraction and clean-up procedures in one step especially for solid and semi-solid samples [5,6]. Due to its simplicity and flexibility, MSPD has been widely applied in the analysis of food, environmental, and biological matrices [3]. In recent years, modifications of the classical MSPD have been reported to refine the original method. In the ultrasonic-assisted MSPD, ultrasonication was performed on the MSPD column for speeding the process and improving the extraction yield [7,8]. Additionally, vortex and homogenization have been reported to replace the column elution and blending in the classical MSPD, respectively, to simplify the procedure of preparation [9,10,11,12,13,14]. Another method for the replacement of column was the magnetically assisted MSPD, in which magnetic ion liquid [15] or particles [16] were used as dispersants. Thus, analytes could be simply extracted by magnetic isolation. Additionally, micro/mini-MSPD, which reduced the sample amount in the protocol, was a good choice for reducing the consumption of sample and material [17,18,19,20,21,22,23,24] and improving the greenness of MSPD [25]. These improved methods have promoted the development of sample preparation technology for solid and semi-solid sample.

Homogenous liquid-liquid extraction (HLLE) is an alternative method to the traditional liquid-liquid extraction. In HLLE, a mixture of water and water-miscible solvent is applied for the liquid extraction, which is triggered to form into two separate phases after the introduction of a phase separation agent or condition [26,27,28,29]. Acetonitrile (ACN)-water-based protocol is in the hot area of HLLE studies, particularly the use of salt as a phase separation agent combined with dispersive solid-phase extraction (d-SPE), has been developed into the popular QuEChERS method [30,31]. Compared with traditional liquid-liquid extraction, HLLE shows the advantage of extraction over a wider polarity range. Furthermore, ACN is compatible with chromatography systems; this means that the obtained extract could be directly injected without additional operation of solvent exchange. These enable HLLE to be widely applied in the analysis of multiple analytes in complex matrices [26,27,28,31].

In the present work, we combined the principles of MSPD and HLLE to develop a novel sample preparation method named micro salting-out assisted MSPD (μ-SOA-MSPD). Impacts of its parameters were systematically investigated, and the proposed method was demonstrated to be simple, rapid, and effective with combining advantages of both MSPD and HLLE. Bisphenol compounds, a group of widespread environmental contaminants that can potentially pollute honeybee products [32,33], were successfully determined in bee pollen matrix by using the μ-SOA-MSPD and HPLC-fluorescence detection. To the best of our knowledge, this is the first report of HLLE-modified MSPD, and also the first report on the analysis of bisphenols contaminant in bee pollen sample.

## 2. Results and Discussion

### 2.1. Micro Salting-Out Assisted MSPD

**Figure 1:**
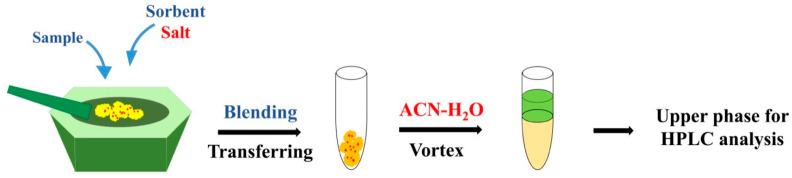
Schematic presentation of micro salting-out assisted matrix solid-phase dispersion (μ-SOA-MSPD).

The schematic procedure of the proposed µ-SOA-MSPD method is shown in Figure 1. Firstly, the sample is blended with salt and sorbent by way of the classical MSPD methodology. According to the principle of MSPD, this step is designed for the disruption of solid sample by using the shearing and grinding force in the blending operation [5]. Afterwards, the blended materials are transferred into a tube and vortexed with solution of ACN-H_2_O mixture. Under vortexing, salt is dissolved into the extractant, triggering the phase separation of ACN from its aqueous solution. After short-time centrifugation to make the phase separation clear, analytes are partitioned into the upper ACN phase with high efficiency. Finally, aliquot of the ACN phase is collected and analyzed by HPLC system.

The unique aspect of this μ-SOA-MSPD method is the introduction of salting-out induced phase separation into the procedure, which makes the HLLE simultaneously accomplished in the MSPD process. As a result, analytes are directly partitioned into the upper ACN phase in this modified MSPD method, which enables the concentration of analytes and provides a substantial clean-up effect as the majority of the matrix is isolated into the lower H_2_O phase. Furthermore, the sorbent material used in the blending step could provide an additional clean-up effect similar to the dispersive solid-phase extraction (d-SPE) [30]. Based on these clean-up effects resulting from the phase partition and the d-SPE behavior of sorbent, the cartridge generally used in the classical MSPD is eliminated in the proposed method. This means that the column wash and elution, the most solvent- and time-consuming steps, are also omitted. Thus, the proposed method possesses the merit of a much simpler procedure, as well as less consumption of time, labor, and organic solvents. These would make the modified MSPD procedure greener than the classical one according to the idea of green analytical chemistry [34].

### 2.2. Salting-Out Parameters

**Figure 2:**
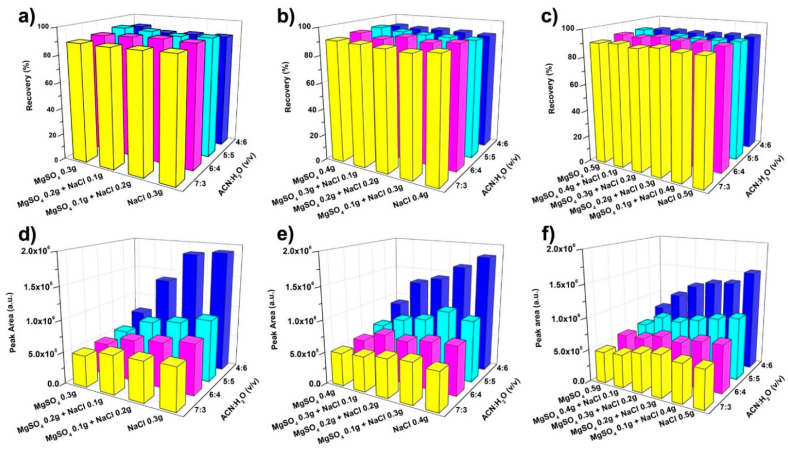
Effects of salts and ACN-H_2_O mixture on the calculated recovery (**a**–**c**) and signal response (**d**–**f**) of bisphenol A (BPA). The total mass of salts were 0.3 g (**a**,**d**), 0.4 g (**b**,**e**), and 0.5 g (**c**,**f**). Mean values of triplicate experiments are presented.

Salt is designed as both the dispersant for the disruption of sample and the phase separation agent in the following partition performance. MgSO_4_ and NaCl were investigated, due to their high efficiency for the phase separation of ACN-H_2_O mixture [29,35]. Application of MgSO_4_ in ACN-H_2_O-based HLLE has shown high extraction yields for compounds with a wide polarity range due to the large volume of separated ACN phase. As regards the NaCl, it provides a relatively small volume of ACN phase, and thus a higher signal response for the target compounds. The effects of salts and ACN-H_2_O mixture on the calculated recovery and the signal response of the analytes are compared in Figure 2.

High recovery values for both bisphenol A (BPA) and bisphenol B (BPB) were observed in extensive salting-out conditions. As shown in Figure 2a–c, the calculated recoveries of BPA were in the range between 88.08% and 93.89%. Under the same recipe of salts, recovery of BPA was slightly increased as the concentration of ACN in the ACN-H_2_O mixture increased from 4:6 to 7:3 (*v*/*v*). Meanwhile, with the same ACN-H_2_O solution, the recipe of salts did not show significant effect on the recovery values of BPA. For instance, under the concentration of 4:6 (*v*/*v*), as the recipe of salts changed from 0.5 g of MgSO_4_ to 0.5 g of NaCl, recovery of BPA varied slightly from 90.43% to 90.71%.

Different from the results on recovery, the signal response of bisphenols was significantly affected by the salts and the ACN-H_2_O mixture (Figure 2d–f). Under the same recipe of salts, the volume of upper phase decreased as the concentration of ACN in the ACN-H_2_O mixture was reduced. As a result, concentration of analytes in the ACN phase could be significantly increased. For example, with the presence of 0.3 g of NaCl, as the concentration of ACN was reduced from 7:3 to 4:6 (*v*/*v*), the signal response of BPA increased to about triple. On the other hand, since NaCl resulted into smaller volume of ACN phase than MgSO_4_ [35,36], signal response of BPA became much higher when the recipe of salts changed from total MgSO_4_ to MgSO_4_-NaCl mixture, and then to total NaCl. Typically, in the ACN-H_2_O mixture of 4:6 (*v*/*v*), the signal response of BPA obtained by 0.3 g of MgSO_4_ was only about 40% of that using the same mass of NaCl. It is important to notice that increasing the mass of salts also led to the increase of the volume of ACN phase, and in consequence reducing the signal. When the concentration of ACN was 4:6 (*v*/*v*), as the mass of NaCl increased from 0.3 g to 0.5 g (Figure 2d–f), signal response of BPA decreased about 20%. It should be pointed out that the effects of salts and ACN-H_2_O mixture on BPB (Figure S1 in Supplementary Materials) were similar to those on BPA. Based on the above results, 0.3 g of NaCl and ACN-H_2_O mixture with concentration of 4:6 (*v*/*v*) were selected as the optimal salting-out conditions, as they provided the highest signal response.

### 2.3. Clean-Up Performance

**Figure 3:**
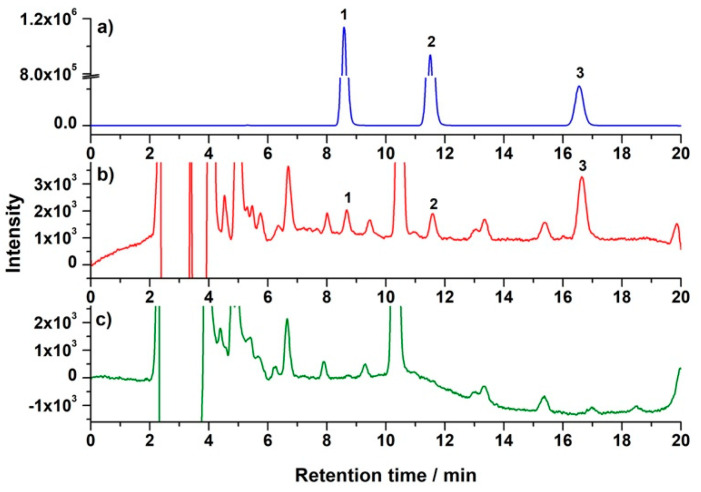
Representative HPLC-fluorescence detector (FLD) chromatograms of (**a**) bisphenol standards, (**b**) spiked bee pollen sample, and (**c**) blank bee pollen sample. Peak 1: bisphenol A (BPA); 2: bisphenol B (BPB); 3: internal standard (IS). The spiked concentrations in (**b**) were 60 μg/kg and 80 μg/kg for BPA and BPB, respectively.

**Figure 4:**
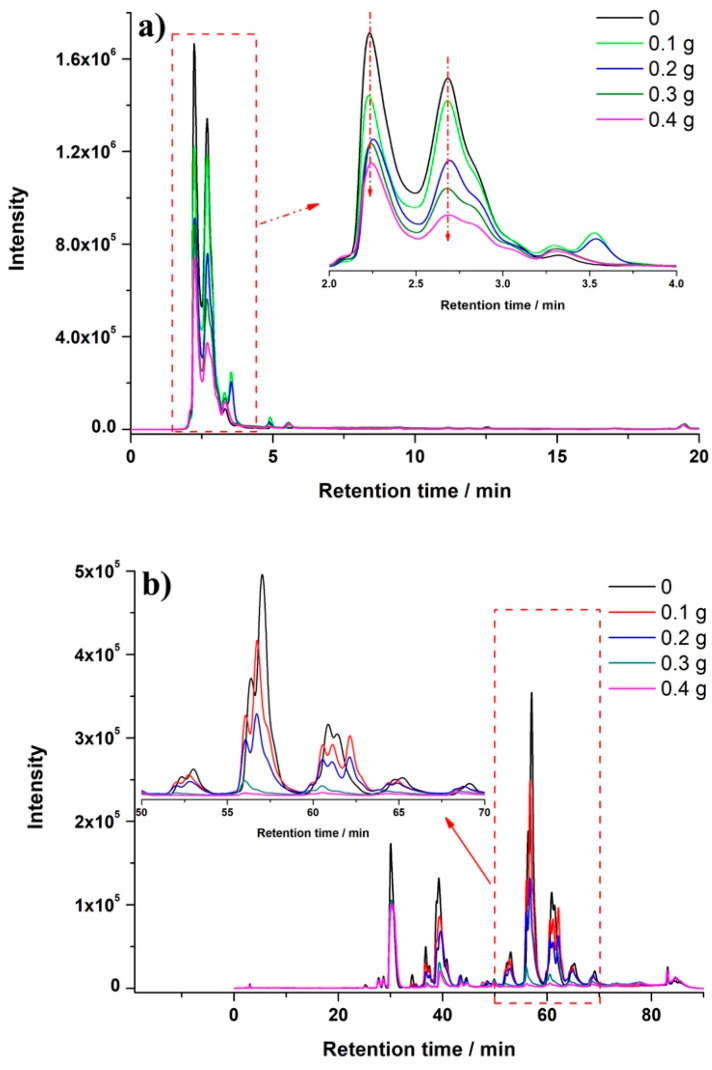
Representative HPLC-DAD chromatograms of extract under different masses of PSA. (**a**) Separation was performed for the analysis of bisphenols (λ = 210 nm), (**b**) separation was performed for the analysis of phenolic compounds (λ = 280 nm).

In this μ-SOA-MSPD method, sorbent could be introduced into the blending step to improve clean-up performance. Bisphenol analytes were separated in reversed-phase HPLC and detected by fluorescence detector (FLD) (Figure 3). To better illustrate the clean-up effect, UV-Vis signal was recorded by diode array detector (DAD). As shown in Figure 4a, introduction of PSA in the blending exhibited remarkable reduction of matrix peaks. It was noticed that the clean-up behavior was happened in the lower retention time, which implied that relative polar compounds in the matrix might be removed by PSA. This was consistent with the reported results using PSA-based solid-phase extraction, that the PSA pipette column showed an excellent retention of phenolic compounds in bee pollen [37]. Therefore, the HPLC condition for the separation of phenolic compounds [29,38] was implemented to further demonstrate the clean-up ability. As indicated in Figure 4b, intensive matrix peaks were observed in the chromatogram of extract prepared in the absence of sorbent. Interestingly, these peaks were dramatically reduced with the addition of PSA in the blending. As the mass of PSA increased to 0.4 g, peaks area located from RT 50 min to 70 min were significantly reduced to only 4% of that obtained without addition of PSA. These DAD results demonstrated the excellent clean-up ability when the sorbent was added in the blending process. Meanwhile, for the fluorescence detection of bisphenols, the HPLC chromatogram was selective and clear enough, and no additional improvement in FLD was observed when the PSA was introduced (Figure S2). Therefore, in the case of bisphenols determination in bee pollen using HPLC-FLD, μ-SOA-MSPD could be performed by simply blended with salt without the presence of PSA.

### 2.4. Method Validation

Seven levels of calibration curves ranging from 0.002 μg/mL to 0.4 μg/mL were applied for the quantification. Good linearity with correlation coefficients of >0.9995 were achieved. The limits of detection (LOD, S/N = 3) for BPA and BPB in bee pollen sample were 20 μg/kg and 30 μg/kg, respectively; and the limits of quantification (LOQ, S/N = 10) were 60 μg/kg and 80 μg/kg, respectively. With spiked level of LOQ, concentration of analytes in the final extractant was about 0.008 μg/mL. The accuracy and precision were investigated in blank bee pollen sample spiked at three levels (1×LOQ, 5×LOQ, and 10×LOQ). Results of the calculated recoveries and RSDs are shown in Table 1. The average recoveries for BPA and BPB were between 87.70% and 94.64%, and between 83.03% and 89.59%, respectively. The precisions were in the range of 1.76% to 5.21%, and 2.07% to 5.45% for BPA and BPB, respectively. All the results were in the acceptable range according to the AOAC [39]. In addition, eleven commercial rape bee pollen samples collected from local markets were analyzed using the proposed method. The results showed that no bisphenols were detected in these samples. More samples using different species of bee pollen should be investigated to reveal the contamination level of bisphenols. In addition, improving the enriching effect of the sample preparation method would be helpful for detecting ultra-low level of bisphenols in bee pollen. We observed lower volumes of extractant phase when a lower mass of salts and lower concentration of ACN in the ACN-H_2_O mixture were used in the μ-SOA-MSPD. This would be valuable for the further development of micro-extraction methodology. These studies are under way.

## 3. Materials and Methods

### 3.1. Materials

ACN (HPLC grade) was obtained from Merck (Darmstadt, Germany). Standards of BPA, BPB, and 4, 4′-cyclohexylidenebisphenol (internal standard, IS) were purchased from Aladdin (Shanghai, China). MgSO_4_ was from Sinopharm Chemical Reagent Co., Ltd. (Shanghai, China) and NaCl was from Xilong Science Co., Ltd. (Guangdong, China). The PSA was obtained from Sepax (Suzhou, China). Ultrapure water (18.2 MΩ) was used through this article. Rape (*Brassica campestris*) bee pollen used for the method development was collected in Hubei, China. Commercial rape bee pollen samples applied in the method application were purchased from local markets. Stock solution of standards were prepared in ACN at the concentration of 1 mg/mL. Working standard solutions were prepared by further diluted with ACN. All standard solutions were stored at 4 °C until used.

### 3.2. Optimal Micro Salting-Out Assisted MSPD

A bee pollen sample (0.1 g) and NaCl (0.3 g) were blended together for 30 s, and then the materials were transferred into a 10 mL tube. After the addition of 4 mL of ACN-H_2_O (4:6, *v*/*v*) solution, the mixture was vortexed for 1 min. Then the mixed solution was centrifuged at 6000 rpm for 5 min to make the phase separation clear. Aliquot of the upper phase was collected and analyzed by HPLC.

### 3.3. Optimization of Salting-Out Parameters

A bee pollen sample (0.1 g) was spiked with 10 μL of standards solution (100 μg/mL of BPA and BPB) and 10 μL of IS solution (100 μg/mL), then stood for 30 min. The spiked sample with different masses of salts (MgSO_4_ and NaCl with total amount from 0.3 g to 0.5 g) were blended together, then the materials were transferred into a 10 mL tube. After the addition of 4 mL of different ACN-H_2_O solutions (4:6, 5:5, 6:4, and 7:3, *v*/*v*), the mixture was vortexed for 1 min. Then the mixed solution was centrifuged at 6000 rpm for 5 min, and an aliquot of the upper phase was collected and analyzed by HPLC.

### 3.4. Clean-Up Effect of PSA in the Micro Salting-Out Assisted MSPD

The spiked bee pollen sample with different masses of PSA (0.1, 0.2, 0.3, and 0.4 g) and 0.3 g of NaCl were blended together, then the materials were transferred into a 10 mL tube. After the addition of 4 mL of ACN-H_2_O solution (4:6, *v*/*v*), the mixture was vortexed for 1 min. Then the mixed solution was centrifuged at 6000 rpm for 5 min, and an aliquot of the upper phase was collected and analyzed by HPLC.

### 3.5. HPLC Analysis

The HPLC system consisted of LC-20AT pump, SIL-20AC autosampler, CTO-20AC column oven, SPD-M20A DAD and RF20-AXL FLD.

For the analysis of bisphenols, an InertSustain (Shimadzu GL, Tokyo, Japan) C18 column (4.6 mm × 250 mm, 5 μm) was used for the separation. The mobile phase consisted of water (solvent A) and ACN (solvent B). Chromatographic conditions were carried out as follows: 47% solvent B at 0–14 min, 47% to 80% at 14−17 min, and maintained at 80% at 17–20 min, then post-run with 3 min for back to 47% and maintained at 47% for 10 min. The flow rate was 1 mL/min, injection volume was 10 µL, and the column temperature was 35 °C. The detection wavelength of DAD was 210 nm, and the excitation and emission wavelength of FLD were 270 nm and 305 nm, respectively.

Phenolic compounds were separated based on the previously reported method [29,38]. A WondaCract ODS-2 (Shimadzu GL, Tokyo, Japan) C18 column (4.6 mm × 150 mm, 5 µm) was applied for the separation. The mobile phase consisted of water with 0.1% (*v*/*v*) acetic acid (solvent A) and methanol (solvent B). Chromatographic conditions were carried as follows: 15–40% solvent B at 0–30 min, 40–55% at 30–65 min, 55–62% at 65–70 min, 62–100% at 70–80 min, then back to 15% at 80–85 min and maintained at 15% for 5 min. The flow rate was 0.8 mL/min, injection volume was 10 μL, and the column temperature was 35 °C. The detection wavelength of DAD was 280 nm.

### 3.6. Method Validation

Seven-level standard curves were prepared containing BPA (0.002, 0.01, 0.02, 0.05, 0.1, 0.2, and 0.4 μg/mL), BPB (0.002, 0.01, 0.02, 0.05, 0.1, 0.2, and 0.4 μg/mL), and IS (0.1 μg/mL). The ratio of peak area (analyte/IS) versus the ratio of weight (analyte/IS) was used to construct the analytical curves. The y-intercept was set to zero and a linear fit was performed. Blank bee pollen sample with the absence of analytes was used for the method validation. The limit of detection (LOD) and limit of quantification (LOQ), defined as 3×S/N (signal/noise) and 10×S/N, respectively, were investigated in spiked blank bee pollen samples, which were prepared as described in Section 3.2. Accuracy and precision studies were estimated by analyzing blank samples spiked at three levels (1×LOQ, 5×LOQ, and 10×LOQ), which were prepared as described in Section 3.2. Recovery was used to express the accuracy, and the precision was determined as relative standard deviation (RSD) to the mean recovery in repeatability (intra-day, *n* = 6) and intermediate precision (inter-day, three consecutive days, *n* = 18) analysis.

## 4. Conclusions

In summary, a new sample preparation method, μ-SOA-MSPD, was developed for the analysis of bisphenol contaminants in bee pollen. Salt and the ACN-H_2_O mixture could be designed to achieve better recovery and signal response. In addition, the introduction of sorbent in the blending step showed excellent removal of matrix components. The proposed method was fully validated, and the results indicated that it was suitable for the reliable and sensitive detection of BPA and BPB residues in spiked bee pollen. The proposed method was simple, rapid, and provided advantages in saving time, labor, and solvents. Since the flexibility of MSPD technology in different matrices, this modified MSPD method would be valuable for the fast sample preparation of other solid and semi-solid samples.

## Figures and Tables

**Table 1:** Accuracy and precision of the proposed method at three spiked levels.

Analytes	Spiked Levels (μg/kg)	Intra-Day						Inter-Day	
		Day 1		Day 2		Day 3			
		Recovery (Mean ± SD, %, *n* = 6)	RSD (%, *n* = 6)	Recovery (Mean ± SD, %, *n* = 6)	RSD (%, *n* = 6)	Recovery (Mean ± SD, %, *n* = 6)	RSD (%, *n* = 6)	Recovery (Mean ± SD, %, *n* = 18)	RSD (%, *n* = 18)
BPA	60	92.70 ± 4.13	4.46	90.19 ± 4.41	4.89	94.64 ± 3.59	3.79	92.51 ± 4.24	4.59
	300	89.13 ± 1.57	1.76	88.44 ± 2.75	3.11	87.70 ± 4.57	5.21	88.43 ± 3.07	3.47
	600	89.04 ± 3.08	3.46	90.16 ± 2.92	3.24	88.30 ± 2.25	2.55	89.16 ± 2.81	3.15
BPB	80	85.14 ± 2.31	2.71	89.59 ± 4.46	4.98	85.25 ± 4.65	5.45	86.66 ± 4.28	4.94
	400	84.29 ± 2.26	2.68	83.18 ± 2.20	2.64	83.46 ± 2.50	3.00	83.64 ± 2.24	2.68
	800	83.03 ± 1.72	2.07	83.26 ± 1.69	2.03	83.68 ± 2.61	3.12	83.32 ± 1.99	2.39

## Data Availability

The data presented in this study are available on request from the corresponding author.

## Supplementary Materials

The following are available online, Figure S1: Effects of salts and ACN-H_2_O mixture on the calculated recovery and signal response of BPB. Figure S2: Representative HPLC-FLD chromatograms of extract under different mass of PSA.

## Sample Availability

Samples of the compounds are not available from the authors.
