# Supplementary material for: Micro Salting-Out Assisted Matrix Solid-Phase Dispersion: A Simple and Fast Sample Preparation Method for the Analysis of Bisphenol Contaminants in Bee Pollen
Source: Molecules. 2021 Apr 18;26(8):2350. doi: 10.3390/molecules26082350 (PMC8074014; doi:10.3390/molecules26082350)
Supplement: Supplementary file 1 [file molecules-26-02350-s001.zip › molecules-1170236-supplementary.pdf]

molecules

MDPI

Supplementary Materials

Micro salting-out assisted matrix solid-phase dispersion: A simple and fast sample preparation method for the analysis of bisphenol contaminants in bee pollen

Jianing Zhang [1], Fengjie Yu [1], Yunmin Tao [1], Chunping Du [2,3], Wenchao Yang [1,2,3], Wenbin Chen [2,3,*] and Xijuan Tu [2,3,*]

[1] College of Food Science, Fujian Agriculture and Forestry University, Fuzhou 350002, China.
[2] College of Bee Science, Fujian Agriculture and Forestry University, Fuzhou 350002, China.
[3] College of Animal Sciences, Fujian Agriculture and Forestry University, Fuzhou 350002, China.
* Correspondence: wbchen@fafu.edu.cn (W.C.); xjtu@fafu.edu.cn (X.T.)

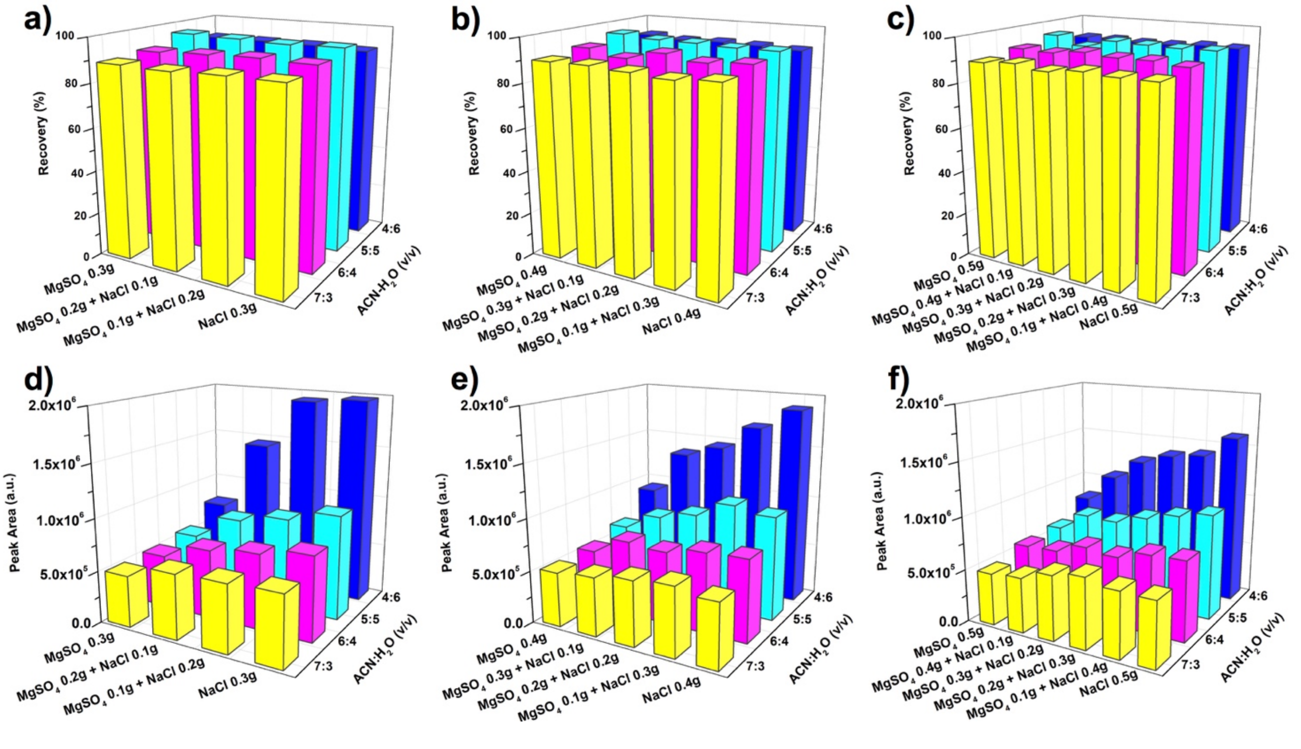

Figure S1. Effects of salts and ACN-H_2O mixture on the calculated recovery (a, b, and c) and signal response (d, e, and f) of bisphenol B (BPB). The total mass of salts were 0.3 g (a and d), 0.4 g (b and e), and 0.5 g (c and f). Mean values of triplicate experiments were presented.

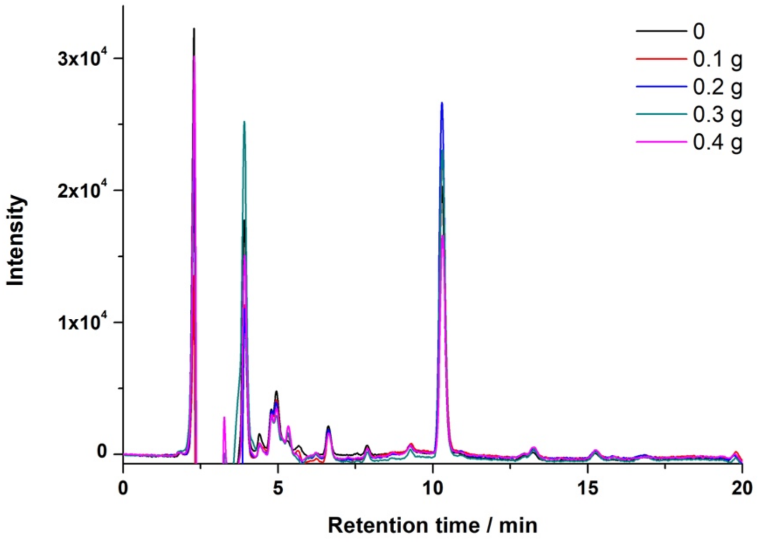

Figure S2. Representative HPLC-FLD chromatograms of extract under different masses of PSA. Separation was performed for the analysis of bisphenols; the excitation and emission wavelength of FLD were 270 nm and 305 nm, respectively.
